# Activation of Human Platelets by *Staphylococcus aureus* Secreted Protease Staphopain A

**DOI:** 10.3390/pathogens11111237

**Published:** 2022-10-26

**Authors:** Amie K. Waller, Katie Birch, Jonathan M. Gibbins, Simon R. Clarke

**Affiliations:** 1 School of Biological Sciences, University of Reading, Whiteknights, Reading RG6 6EX, UK; 2 Institute for Cardiovascular and Metabolic Research, University of Reading, Whiteknights, Reading RG6 6EX, UK

**Keywords:** *Staphylococcus aureus*, platelets, protease, staphopain, PAR-1, PAR-4

## Abstract

Infection by *Staphylococcus aureus* is the leading cause of infective endocarditis (IE). Activation of platelets by this pathogen results in their aggregation and thrombus formation which are considered to be important steps in the development and pathogenesis of IE. Here, we show that a secreted cysteine protease, staphopain A, activates human platelets and induces their aggregation. The culture supernatant of a *scpA* mutant deficient in staphopain A production was reduced in its ability to trigger platelet aggregation. The platelet agonist activity of purified staphopain A was inhibited by staphostatin A, a specific inhibitor, thus implicating its protease activity in the agonism. In whole blood, using concentrations of staphopain A that were otherwise insufficient to induce platelet aggregation, increased binding to collagen and thrombus formation was observed. Using antagonists specific to protease-activated receptors 1 and 4, we demonstrate their role in mediating staphopain A induced platelet activation.

## 1. Introduction

*Staphylococcus aureus* is a major pathogen responsible for a significant burden on human morbidity and mortality. If *S. aureus* enters the bloodstream, the consequent bacteremia can cause complications such as endocarditis and sepsis. The pathogen can activate platelets, forming platelet-bacteria aggregates that become deposited on exposed sub-endothelial extracellular matrix such as collagen, in places such as the heart valves, thus compromising their function [1,2]. Similarly, septic emboli can form, migrate through the circulation to the brain and cause a stroke. In sepsis, thrombocytopenia can occur as a consequence of platelet consumption, resulting in a poor prognosis for the patient, indeed the severity of thrombocytopenia correlates with the rate of mortality [3,4]. Platelets from *S. aureus* endocarditis patients display heightened reactivity to physiological agonists [5] and activation induced by the infectious milieu is generally assumed to cause thrombocytopenia, as activated platelets aggregate and are deposited in the microvasculature, resulting in disseminated intravascular coagulation, multi-organ failure [6,7] and loss of haemostatic function [8]. In addition to their haemostatic function, platelets contribute to the initiation and coordination of effective immune responses to infection [9,10].

The ability of *S. aureus* to activate platelets has been attributed to the presence of surface proteins known as microbial surface components recognising adhesive matrix molecules (MSCRAMMs), which bind plasma proteins such as fibrinogen and von Willebrand factor, causing activation via the plasma protein’s cognate receptors on the platelet [2,11,12,13,14,15,16,17,18,19,20,21]. While MSCRAMMs are important factors in this aspect of the host–pathogen interaction, *S. aureus* also produces a wide variety of extracellular molecules such as toxins and proteases that interact with the host. *S. aureus* α-haemolysin (Hla) is a potent activator of platelets [22], but its relevance to endocarditis has been questioned as strains which produce high quantities of the toxin are not associated with the disease; indeed this may be related to its ability to lyse platelets at higher concentrations [23]. Activation of platelets in vivo by α-haemolysin does however cause formation of dynamic microthrombi which accumulate in the liver sinusoids and kidney glomeruli, causing multi-organ disfunction [7]. Conversely, *S. aureus* strains that produce proteases are more likely to cause the disease. The pathogen produces 4 main proteases, a metalloprotease (aureolysin, Aur), a serine protease (V8, SspA) and two cysteine proteases (staphopain A (ScpA) and staphopain B (SspB)), which have multiple effects on host cells and molecules [24,25].

In this study, we show that staphopain A, an intensively secreted protease of *S. aureus* which is conserved amongst clinical isolates, induces platelet activation and that this occurs via protease-activated receptors 1 and 4 (PAR-1 and -4). At concentrations of staphopain A that are insufficient to cause platelet aggregation, it potentiates the ability of the platelet to become activated by collagen. Thus, staphopain A is able not only to directly activate platelets but can also prime them, increasing activation stimulated by collagen, a normal physiological agonist to which they can be exposed upon damage to the endothelium. 

## 2. Materials and Methods

### 2.1. Reagents

Bovine thrombin, Fluorescein isothiocyanate (FITC), dialysis tubing, wortmannin, TFLLR-NH_2_ and AYPGKF-NH_2_ were obtained from Sigma (Poole, UK). Bisindolylmaleimide I (Bis-1) and primary 4G10 antibody were purchased from Merck Millipore (Darmstadt, Germany). RWJ56110 and TCY-NH_2_ were purchased from R&D Systems (Minneapolis, MN, USA). Primary FITC labeled PAC-1 and Cy5 labelled anti-CD62P were purchased from BD Biosciences (New Jersey, NJ, USA). Staphostatin A was purchased from Biocentrum (Krakow, Poland). Fast flow DEAE and CF sepharose columns were purchased from GE Healthcare (Buckinghamshire, UK). Brain heart infusion media was purchased from Oxoid (Basingstoke, UK). All other reagents were from previously described sources [26].

### 2.2. Bacterial Strains Used

Wild type *S. aureus* 8325-4 [27], and mutant strains DU1090 (*hla*) [28], LES12 (*aur*), LES17 (*sspB*), LES22 (*sspABC*) and LES27 (*scpA*) [29] were used. 

### 2.3. Ammonium Sulphate Precipitation of S. aureus Supernatant

300 mL BHI broth was inoculated 1:100 with overnight cultures of *S. aureus* and grown for 6 h. Supernatant was then harvested by centrifugation at 15,000× *g* for 10 min. Ammonium sulphate was added to the supernatant to a final concentration of 3.1 M and left stirring for 1 h at 4 °C. The supernatant was centrifuged at 20,000× *g* for 20 min and the pellet was resuspended in 2 mL of PBS. Ammonium sulphate fractions were then dialysed thoroughly against PBS to remove all ammonium sulphate. Protein concentrations were determined by Bradford assay (Bio-Rad, Hercules, CA, USA). 

### 2.4. Extraction and Purification of Staphopain A

Staphopain A was purified using a previously described method [30,31]. 300 mL BHI broth was inoculated with *S. aureus* DU1090 and grown overnight at 37 °C. The supernatant was then harvested by centrifugation at 15,000× *g* for 20 min. Ammonium sulphate was added to the supernatant to a final concentration of 4.25 M and left stirring for 1 h at 4 °C. The supernatant was centrifuged at 20,000× *g* for 20 min and the pellet was resuspended in 5 mL of 50 mM sodium acetate. Ammonium sulphate fractions were then dialysed thoroughly against 50 mM sodium acetate to remove all ammonium sulphate. The dialysed fractions were then passed through a Fast flow DEAE column to remove any staphopain B. The flow through was collected and ion exchange chromatography was performed using a fast flow CF sepharose column with an increasing gradient of 300 mM sodium chloride. The presence of a single band corresponding to the known size of staphopain A was determined by SDS-PAGE. These fractions were pooled and dialysed against Tyrode’s buffer and lyophilised overnight. The concentration of staphopain A was determined by Bradford assay. 

### 2.5. Preparation of Platelet Rich Plasma

Human blood was obtained from consenting healthy volunteers who were free from drugs known to influence platelet function. This work was approved by the University of Reading Ethics Committee. 45 mL of blood was collected into a syringe containing 5 mL of anti-coagulant 4% (*w*/*v*). Platelet rich plasma (PRP) was prepared by centrifugation at 102× *g* for 20 min. Platelet concentration was adjusted to 4 × 10^8^ platelets/mL and PRP was rested for 30 min at 30 °C prior to experiments. 

### 2.6. Human Washed Platelet Preparation

Human blood was obtained from consenting healthy volunteers in accordance with the principles set out in the Declaration of Helsinki. Ethical approval for all experiments using human blood was obtained from the University of Reading Research Ethics Committee. All donors gave written consent. 45 mL of blood was collected into a syringe containing 5 mL of anti-coagulant 4% (*w*/*v*) sodium citrate and mixed with 7 mL of acid citrate dextrose (ACD) and were prepared by differential centrifugation [20]. Platelets were re-suspended in modified Tyrode’s-HEPES buffer (134 mM NaCl, 0.34 mM Na_2_HPO_4_, 2.9 mM KCl, 12 mM NaHCO_3_, 20 mM HEPES, 5 mM glucose and 1 mM MgCl_2_, pH 7.3) and rested for 30 min at 30 °C prior to experiments.

### 2.7. Light Transmission Aggregometry

Human washed platelet or PRP were prepared as described. Platelet samples were incubated for 30 min at 37 °C with the antagonists indicated followed by stimulation by agonists. Aggregation was measured in an optical aggregometer (Chronolog), stirring whilst at 37 °C. Percentage aggregation was calculated by dividing the maximum aggregation of samples by the maximum aggregation achieved by the given agonist alone [32] Aggregation was measured for 300 s.

### 2.8. Flow Cytometry 

5 µL of washed platelets (4 × 10^8^ cells/mL) or PRP, was prepared as mentioned previously, and incubated with 5 µL of PAC-1 antibody or 1 µL of anti-CD62P. PRP was then incubated with various concentrations of staphopain A, staphostatin A, or thrombin (1 unit/mL). Samples were fixed at room temperature for 10 min in 1% paraformaldehyde. Samples were diluted with Tyrode’s buffer to a final volume of 500 µL. Fluorescence intensity of the sample was measured using a BD Accuri™ C6 flow cytometer. 10,000 events within the platelet gate as determine by forward and side scatter were measured per sample.

### 2.9. In Vitro Thrombus Formation

Whole citrated blood was incubated with a lipophilic dye, 3,3-dihexyloxacarbocyanine iodide (DIOC6) for 10 min and perfused through collagen-coated (400 μg mL^−1^) Vena8™Biochip (Cellex, Dublin, Ireland) at a shear rate of 20 dynes/cm^2^. Z-stack images (epifluorescence) of forming thrombi were taken every 30 s using a Nikon eclipse (TE2000-U) microscope and thrombus fluorescence intensity was analyzed using Slidebook™5 software (Intelligent Imaging Innovations, Denver, CO, USA).

### 2.10. Statistical Analysis

Statistical analysis was performed in GraphPad Prism version 9.4.1 (GraphPad Software, La Jolla CA, USA). Data was determined as normally distributed using a Shapiro–Wilk test. For normally distributed data sets, significance was assessed using unpaired T-tests. For non-normally distributed data sets, a Wilcoxon Signed Rank test was used to determine significance (indicated in figure legend). *p* < 0.05 was regarded as significant.

## 3. Results

### 3.1. Staphopain A Causes Platelet Activation

**Figure 1:**
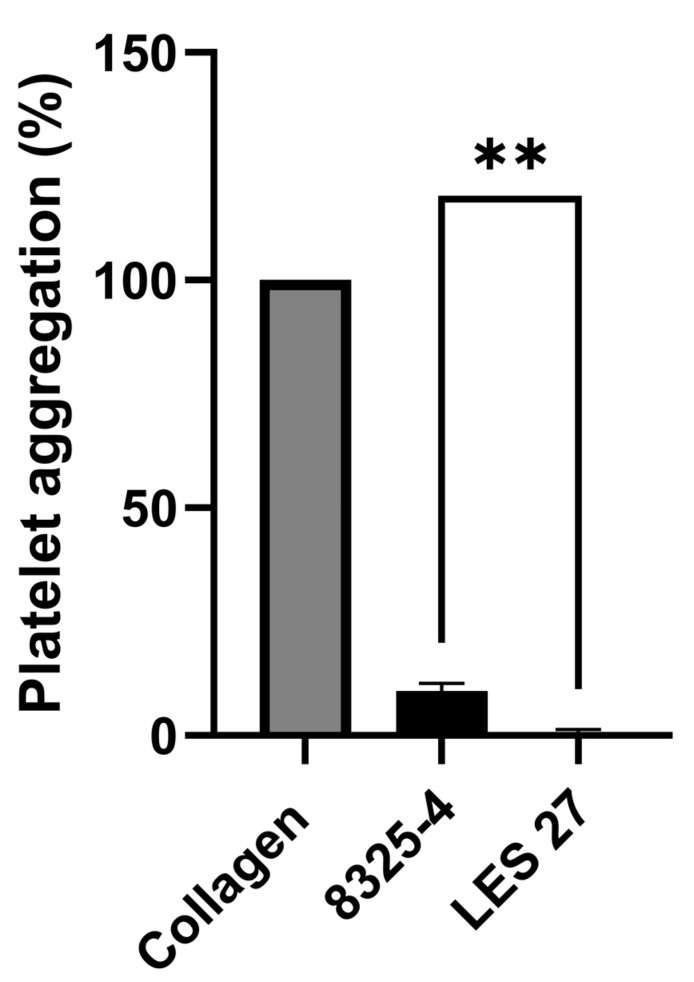
Supernatant from *S. aureus* induces platelet aggregation. Aggregation of washed human platelets (4 × 10^8^ platelets/mL) stimulated with precipitated supernatant of *S. aureus* 8325-4 and LES27 (*scpA*). Aggregation was measured as change in light transmission for 300 s. Collagen agonism was used to standardise responses between platelet donors. Mean values ± SEM, *n* = 3, ** *p* < 0.01.

In order to determine whether *S. aureus* proteases were able to induce activation of human platelets, the exoproteins of stationary phase cultures of 8325-4 and isogenic protease mutants LES12 (*aur*), LES17 (*sspB*), LES22 (*sspABC*) and LES27 (*scpA*) were precipitated with a range of ammonium sulphate concentrations up to 4.25 M, equivalent of *c.* 80% saturation which is sufficient to precipitate all 4 *S. aureus* proteases [31,33,34]. The ability of the re-suspended precipitates to induce platelet aggregation was subsequently tested. As expected, we observed aggregation corresponding to the presence of Hla (results not shown). Additionally, exoproteins precipitated with 3.1 M ammonium sulphate from the supernatant of *S. aureus* 8325-4, but not LES27 (*scpA*) possessed the ability to induce aggregation of platelets (Figure 1).

The supernatants of LES12 (*aur*), LES17 (*sspB*), LES22 (*sspABC*) (Figure S1) and DU1090 (*hla*), precipitated with 3.1 M ammonium sulphate induced aggregation in a manner indistinguishable from the parental wild type. Addition of ammonium sulphate up to 4.25 M did not reveal the presence further agonist activity (results not shown). Thus we were able to demonstrate that *S. aureus* 8325-4 supernatant possesses staphopain A-dependent platelet aggregatory activity in addition to Hla.

**Figure 2:**
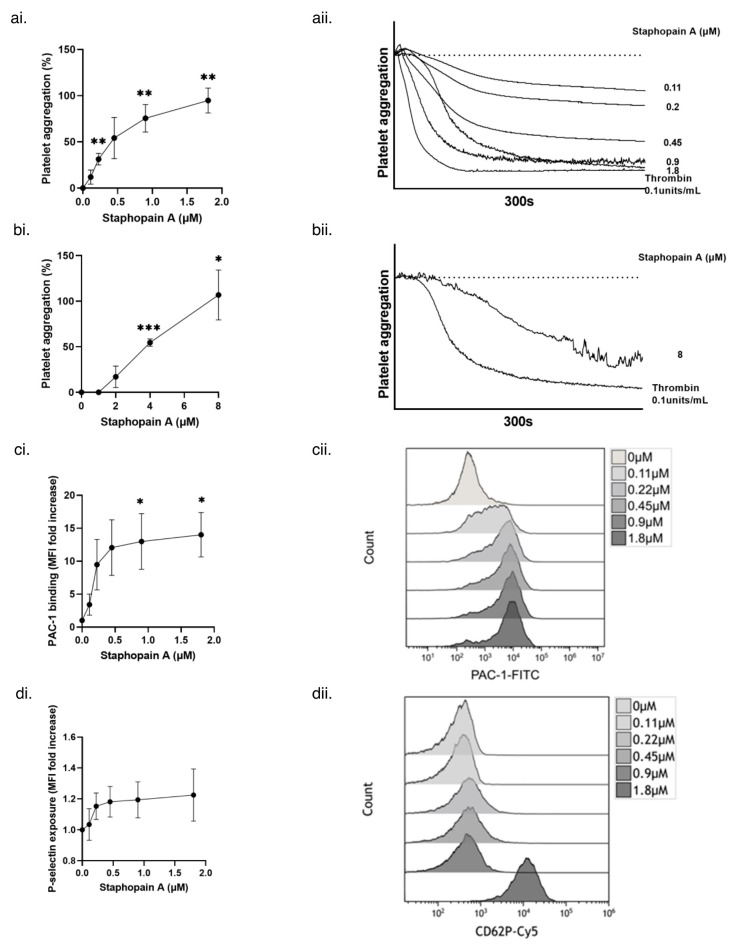
Purified Staphopain A causes platelet aggregation, activation of α_IIb_β_3_ and α-granule secretion. (**ai**) Washed human platelets (4 × 10^8^ cells/mL) were stimulated with increasing concentrations of staphopain A. Aggregation was measured as change in light transmission for 300 s. (**aii**) Representative aggregation traces. (**bi**) Human PRP was stimulated with increasing concentrations of staphopain A. Aggregation was measured as change in light transmission for 300 s. (**bii**) Representative aggregation traces. (**ci**) Washed human platelets (4 × 10^8^ cells/mL) were stimulated with increasing concentrations of staphopain A and incubated with PAC-1 FITC. Samples were run through a BD Accuri^TM^ C6 flow cytometer and median fluorescence was recorded. Data are plotted as median increase in fluorescence when compared to a Tyrode’s buffer only control. (**cii**) Representative histograms. (**di**) Washed human platelets (4 × 10^8^ cells/mL) were stimulated with increasing concentrations of staphopain A and incubated with CD62P Cy5. Samples were run through a BD Accuri^TM^ C6 flow cytometer and median fluorescence was recorded. Data are plotted as median increase in fluorescence when compared to a Tyrode’s buffer only control. (**dii**) Representative histograms. Mean values ± SEM, *n* = 3, * *p* < 0.05, ** *p* < 0.01, *** *p* < 0.005.

Staphopain A was purified to homogeneity from the supernatant (Figure S2) for use in further experiments from *S. aureus* DU1090 (*hla*), in order to eliminate the possibility of trace contamination with Hla. Using platelet aggregometry, staphopain A was able to induce aggregation of washed human platelets Figure 2(ai,aii) and PRP Figure 2(bi,bii), in a dose dependent manner.

Upon activation of platelets, a conformational change in integrin α_IIb_β_3_ occurs, allowing it to bind fibrinogen [35]. P-selectin, an adhesive molecule, also becomes exposed on platelet surfaces as a consequence of α-granule secretion. In order to confirm that platelets were indeed activated by staphopain A, antibodies reactive to P-selectin (anti-CD62P) [36] and the activated form of α_IIb_β_3_ (PAC-1) [37] were used in flow cytometry. In both instances, a dose dependent increase in binding of PAC-1 (Figure 2ci,cii) and anti-CD62P (Figure 2di,dii) occurred. Taken together, these data using defined isogenic *S. aureus* strains, and flow cytometry, demonstrate that *S. aureus* staphopain A induces the activation and consequent aggregation of human platelets.

### 3.2. Staphopain A Protease Activity Is Required for Platelet Activation and Intracellular Signaling

**Figure 3:**
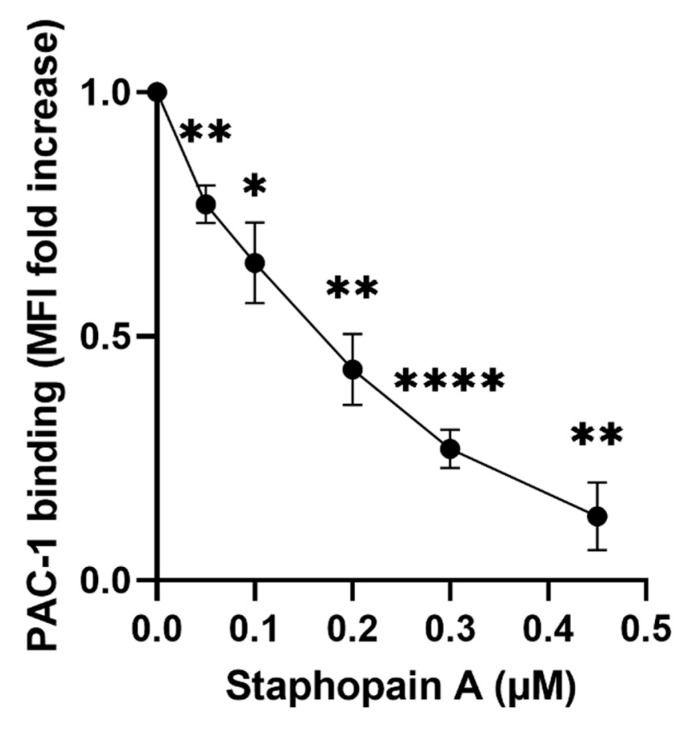
Activation of α_IIb_β_3_ caused by staphopain A is inhibited by staphostatin A. Increasing amounts of staphostatin A was added to staphopain A (0.45μM) and after 5 min incubation PAC-1 FITC was added. Samples were run through a BD Accuri^TM^ C6 flow cytometer and median fluorescence was recorded. Data are plotted as median increase in fluorescence when compared to Staphopain A only. Mean values ± SEM, *n* = 3, * *p* < 0.05, ** *p* < 0.01, **** *p* < 0.0001.

Given that mammalian proteases are well known agonists of platelet activation, it was hypothesised that the protease activity of staphopain A was responsible for the observed ability to activate platelets. Staphostatin A (ScpB) was used to inhibit the activity of the staphopain A. Staphostatin A is an *S. aureus* intracellular inhibitor of staphopain A, which binds the protease in a 1:1 inhibitory complex, blocking its active site and preventing its activity prior to secretion into the extracellular milieu [38,39,40]. Staphostatin A is highly selective for staphopain A and is inactive against trypsin, chymotrypsin, neutrophil elastase, cathepsin G or all of the other *S. aureus* proteases [39]. Following exposure to increasing concentrations of staphostatin A, PAC-1 binding to platelets was measured as a marker of platelet activation. Increasing concentrations of staphostatin A reduced the amount of PAC-1 binding to platelets in a dose-dependent manner (Figure 3), thus the protease activity of staphopain A is responsible for the observed platelet activation.

### 3.3. Staphopain A Potentiates Thrombus Formation under Physiological Flow Conditions

Suboptimal activation of platelets by thrombin enhances their adhesion to collagen via integrin α_2_β_1_ [41]. This is believed to prime platelets to form thrombi on exposed sub-endothelial matrix without directly activating them. Moreover, *S. aureus* increases platelet reactivity in patients with infective endocarditis [5].

**Figure 4:**
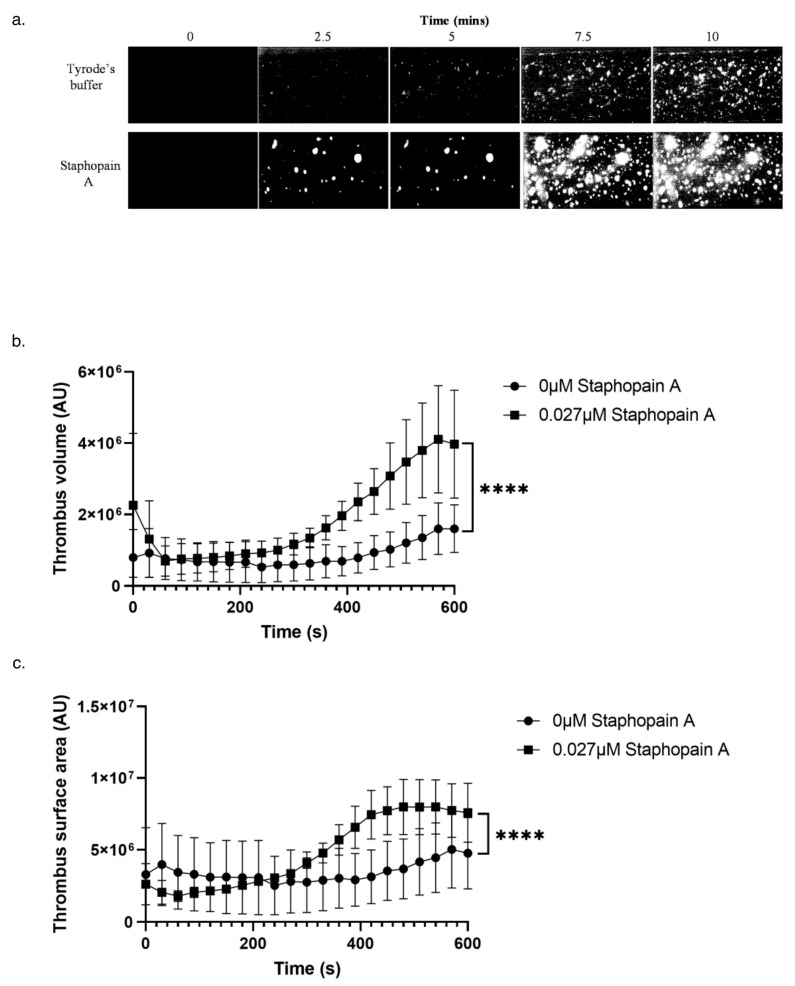
Staphopain A induces thrombus formation at sub-activation concentrations. (**a**) Platelets within whole human blood were labelled with a lipophilic dye DIOC6. Whole blood was then treated with staphopain A (0.027 μM) and then perfused through collagen coated (400 µg/mL) Vena8™Biochip at a flow rate of 20 dynes cm^−2^. Formation of thrombi was recorded using a Z-stack capture every 30 s for 10 min using a Nikon eclipse (TE2000-U) microscope. Thrombus fluorescence volume and surface area was calculated using Slidebook™ 5.0 software. Representative images. (**b**) Volume of thrombi formed over 10 min. (**c**) Surface area of thrombi formed over 10 min. Data were not normally distributed thus a Wilcoxon Signed Rank test was performed to determine significance. Mean values ± SEM, *n* = 3. **** *p* < 0.0001.

To investigate whether staphopain A could potentiate thrombus formation in whole blood and under arterial flow conditions, whole human blood was perfused through collagen coated biochips in the presence of Tyrode’s buffer and staphopain A. In blood pre-treated with staphopain A at a concentration (0.027 μM) which was insufficient to stimulate platelet aggregation, significantly increased thrombus volume and surface area, but not number of thrombi formed, was observed compared to the vehicle treated control (Figure 4a–c). This suggests that initial adhesion to collagen is unaltered but subsequent platelet activation and thrombus growth is enhanced. Mean peak fluorescence was increased by approximately 255% (Figure 4b). 

Potentiation of thrombus formation in whole blood under physiologically relevant flow conditions was consistent with the platelet activation observed in plasma and washed platelets (Figure 2). Furthermore, these data demonstrate that concentrations of staphopain A which might not be sufficient to cause thrombus formation alone, may be sufficient cause septic thrombi to form and/or become larger than would be the case in its absence.

### 3.4. Platelet Activation by Staphopain A Occurs via Protease Activated Receptors 1 and 4 (PAR-1 and -4)

**Figure 5:**
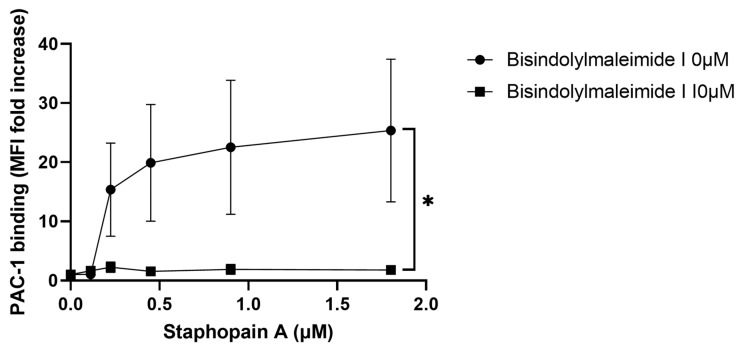
Staphopain A mediated platelet activation occurs via PKC. Whole blood samples were treated with Bisindolylmalemide-I (10 μM) for 30 min. PAC-1 was added before stimulation with various concentrations of staphopain A. Samples were run through a BD Accuri^TM^ C6 flow cytometer and median fluorescence was recorded. Data are plotted as median increase in fluorescence when compared to a Tyrode’s buffer only (no staphopain A) control and represent mean values ± SEM, *n* = 3. * *p* < 0.05.

The activation of human platelets by the mammalian protease thrombin is mediated by cleavage of PAR1 and PAR4. These G-protein coupled receptors induce activation via protein kinase C (PKC) [42,43,44,45,46]. To test whether staphopain A induced activation could be inhibited by blocking PKC, we assessed the ability of a PKC-inhibitory concentration of Bis-1 (10 μM) [47] to prevent activation of integrin α_IIb_β_3_, measured using PAC-1 antibody. Bis-1 mediated PKC inhibition resulted in a significant decrease in α_IIb_β_3_ activation (Figure 5), thus activation of platelets by staphopain A is mediated, at least in part via PKC, possibly downstream of PAR-1 and/or -4.

**Figure 6:**
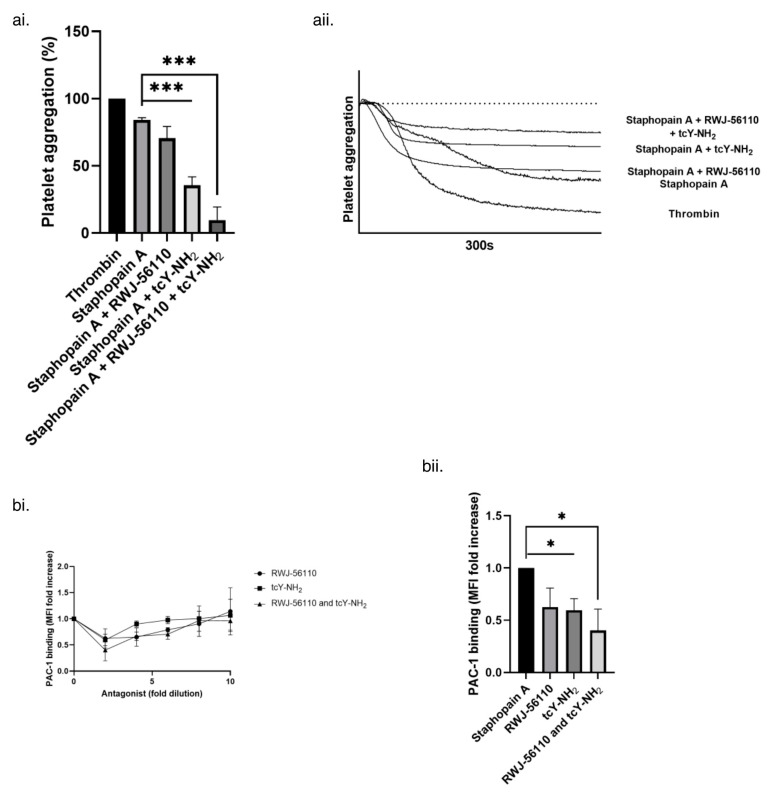
The role of PAR-1 and -4 in staphopain A platelet agonist activity. (**ai**) Washed human platelets (4 × 10^8^ cells/mL) were preincubated for 30 min with RWJ-56110 (100 μM) and/or tcY-NH2 (400 μM) and then stimulated with staphopain A (0.45 μM). Aggregation was measured as change in light transmission for 300 s. (**aii**) Representative aggregation traces. (**bi**) Washed human platelets (4 × 10^8^ cells/mL) were preincubated for 30 min treated with various concentrations of RWJ-56110 and/or tcY-NH2 and then treated with staphopain A (0.45 μM). PAC-1 was added before stimulation with various concentrations of staphopain A. Samples were run through a BD Accuri^TM^ C6 flow cytometer and median fluorescence was recorded. Data are plotted as median increase in fluorescence when compared to a staphopain A only control. (**bii**) Data from (**bi**) shown with RWJ-56110 (50 μM) and/or tcY-NH2 (200 μM) of Mean values ± SEM, *n* = 3. * *p* < 0.05, *** *p* < 0.001.

We next sought to assess the individual roles of PAR-1 and -4 in staphopain A platelet activation. Using RWJ-56110 (PAR-1 antagonist) [48] and tcY-NH_2_ (PAR-4 antagonist) [49], the ability of staphopain A to induce activation was assessed. RWJ-56110 caused a small but reproducible inhibition of aggregation but tcY-NH_2_ caused a much larger decrease in staphopain A induced platelet aggregation. Combining both inhibitors resulted in the largest decrease Figure 6(ai,aii). Similarly PAC-1 binding, a measure of platelet activation, was inhibited by both RWJ-56110 and tcY-NH_2,_ was inhibited by both antagonists Figure 6(bi,bii). Thus it appears that staphopain A activates platelets mainly via PAR-4, but with a contribution from PAR-1 also.

**Figure 7:**
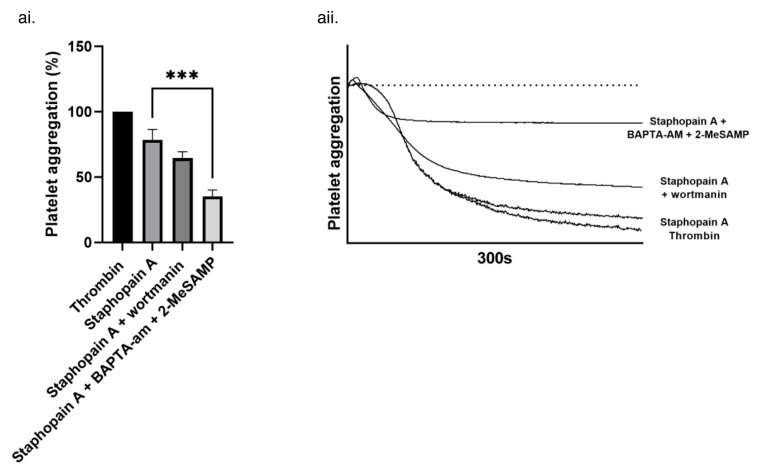
The role of PAR-1 and -4 signalling in staphopain A platelet agonist activity. (**ai**) Washed human platelets (4 × 10^8^ cells/mL) were treated with wortmannin (0.1 μM), BAPTA-AM (20 μM) and 2-MeSAMP (50 μM), and then stimulated with staphopain A (0.45 μM). Aggregation was measured as change in light transmission for 300 s. (**aii**) Representative aggregation traces. Data are plotted as percentage of aggregation and represent mean values + SEM, *n* = 3 *** *p* < 0.001.

To confirm these data, the downstream signaling pathways of PAR-1 and -4 were inhibited. PAR-1 couples to G_i/o_ in human platelets and activates phosphoinositide-3 kinase (PI3K) [50]. PI3K activation regulates integrin α_IIb_β_3_ activation and potentiates the PAR-1 mediated increase in intraplatelet calcium concentration [47]. Wortmannin, a PI3K inhibitor, eliminates these effects downstream of PAR-1 but has no effect on PAR-4 signaling [47,51,52,53]. Aggregation induced by staphopain A was reduced by the addition of wortmannin, indicating a role for PAR-1 signaling (Figure 7).

PAR-4 signalling can be blocked in platelets by dual inhibition of P2Y_12_ purinergic receptor and calcium mobilisation, with no effect on PAR-1 signaling [54]. Using 2-MeSAMP (a P2Y_12_ receptor antagonist) and BAPTA-AM (an intracellular Ca^2+^ chelator), a significant reduction in the ability of staphopain A to induce platelet aggregation was observed (Figure 7).

Taken collectively, these data demonstrate that staphopain A is able to induce platelet activation via PAR-1 and -4. Activation via PAR-4 appears to be greater than the observed PAR-1 effect.

## 4. Discussion

Bacterial host–pathogen interactions are often complex and multifactoral. Our data show that staphopain A causes platelet activation, but we do not conclusively demonstrate a lack of a role for other *S. aureus* proteases in platelet activation, which might potentially be demonstrable under different conditions. Many pathogens, including *S. aureus*, can induce the activation of platelets during bacteraemia, the consequences of which can vary from formation of infected thrombi on heart valves resulting in endocarditis, to disseminated intravascular coagulation and consumption of platelets resulting in thrombocytopenia. Much of the previous research on *S. aureus*-platelet interactions has focused on the role of MSCRAMMs which bind platelets either indirectly via plasma proteins such as fibrinogen, fibronectin and von Willebrand factor, or directly by binding to α_IIb_β_3_. The pathogen possesses significant functional redundancy in this regard, possessing an array of MSCRAMMs which can activate platelets in vitro [2,11,12,13,14,15,16,17,18,19,20,21]. However, to date, only two of these, ClfA and FnBPA, have been shown to be virulence factors in experimental models of infective endocarditis [55,56].

The bacterial extracellular milieu is a cocktail of molecules, some of which are involved in the interaction between pathogens and their hosts. Previous studies of *S. aureus* supernatant molecules have shown that α-hemolysin, Efb, Eap and lipoteichoic acid affect platelets [22,32,57,58]. Similarly, *Porphyromonas gingivalis*, another pathogen of the vasculature, produces proteases that like staphopain A are folded in a papain-like manner, called arginine-specific gingipains RgpB and HRgbA, which activate platelets [59]. Thus, exomolecules produced by pathogens represent an important element in bacteria-platelet interactions.

Staphopain A mediated platelet activation is a novel aspect of the host–pathogen interaction. Hitherto, in vitro studies have shown staphopain A to degrade fibrinogen and collagen [60], release of bradykinin from human kininogens [61], block CXC chemokine receptor 2 (CXCR2) on neutrophils [62] and to induce cell death in epithelial cells [63]. The activities against collagen, fibrinogen and kininogens are proposed to be important in haemostasis. Proteolysis of collagen and fibrinogen correlates with an ability to inhibit the clotting of cell-free blood plasma. Release of bradykinin from kininogens results in vascular leakage and hypotension. Both phenomena which, along with thrombocytopenia, could at least in part account for the disruption of normal haemostasis seen during sepsis.

Some previous in vivo studies on the roles of staphylococcal proteases, which have principally relied on mice in various models, have been inconclusive. This may be explained by α2-macroblobulin and macroglobulin-related proteins, which are inhibitory for staphopains and are present in much higher levels in mice than in humans [64]. However, staphopain A has recently been demonstrated to contribute to colonisation of the lung in a mouse model of pneumonia, as well as host cell death in vitro [63].

In this study, we used antagonists of PAR-1 and -4, and inhibitors of their different signalling pathways to demonstrate that staphopain A mediated platelet activation occurs primarily through PAR-4 and to a lesser extent PAR-1. The PARs are a family of four G protein coupled receptors which act as targets for proteases. Distributed among many different cell types, PARs are substrates for various different proteolytic enzymes. Although most studies on platelet PARs have used thrombin as an agonist, neutrophil granzyme Cathepsin G activates platelets via PAR-4 [65].

In platelets, PAR signalling is well characterised and we have used known inhibitors of these signalling pathways to confirm their role in staphopain A agonism. PAR-1 and -4 exist in a stable complex allowing thrombin to act as a bivalent functional agonist where PAR-4 activity is enhanced by thrombin-PAR-1 interactions [66]. However, activation of either receptor is sufficient to trigger platelet activation. Cleavage of platelet PAR-1 results in rapid signalling across the plasma membrane to internally located G proteins, which culminates in the formation of platelet aggregates [44]. Experiments using thrombin as an agonist show that PAR-1 has a higher affinity for thrombin than PAR-4 and is thus presumed to be the first PAR activated when thrombin is generated at sites of vascular injury [67]. However, PAR-4 signalling is prolonged and is thus proposed to play a role in the late phase of platelet aggregation [68,69]. In this study, we have shown that staphopain A is an agonist principally of PAR-4, but that some platelet activation also occurs via PAR-1.

In addition to their roles in platelet physiology, PARs are involved in inflammation and vascular homeostasis, being present on vascular endothelium, vascular smooth muscle cells, cardiomyocytes and leukocytes [70]. Both PAR-1 and -4 are present in vascular endothelial cells and their activation results in vasodilation [71,72]. In rat models, removal of endothelium from arteries, which is sometimes seen at sites of inflammation and subsequent *S. aureus* infection, can result in PAR-1 mediated direct smooth muscle contraction [73]. PAR-4 activation on cultured human aortic smooth muscle cells supports thrombin production by those cells [74]. In rat venules, thrombin-PAR-4 signalling stimulates proinflammatory effects by inducing leukocyte rolling and adhesion [75]. Activation of PAR-4 on epithelial cells stimulates mesenchymal transition [76]. Thus it is tempting to speculate that staphopain A mediated activation of PAR-4 and possibly PAR-1 might also have effects on other cell types within the vascular system and elsewhere, and thus may represent a novel axis in the host-*S. aureus* interaction. Similarly, PAR-2 and -3, which are not present on human platelets, may also be substrates of *S. aureus* proteases.

## Data Availability

The data that support the findings of this study are available from the corresponding author upon reasonable request.

## Supplementary Materials

The following supporting information can be downloaded at: https://www.mdpi.com/article/10.3390/pathogens11111237/s1, Figure S1: Aggregation of human platelets using strains of *S. aureus*; Figure S2: Purified staphopain A.
