# Supplementary material for: Activation of Human Platelets by Staphylococcus aureus Secreted Protease Staphopain A
Source: Pathogens. 2022 Oct 26;11(11):1237. doi: 10.3390/pathogens11111237 (PMC9696029; doi:10.3390/pathogens11111237)
Supplement: Supplementary file 1 [file pathogens-11-01237-s001.zip › pathogens-1986029-supplementary.pdf]

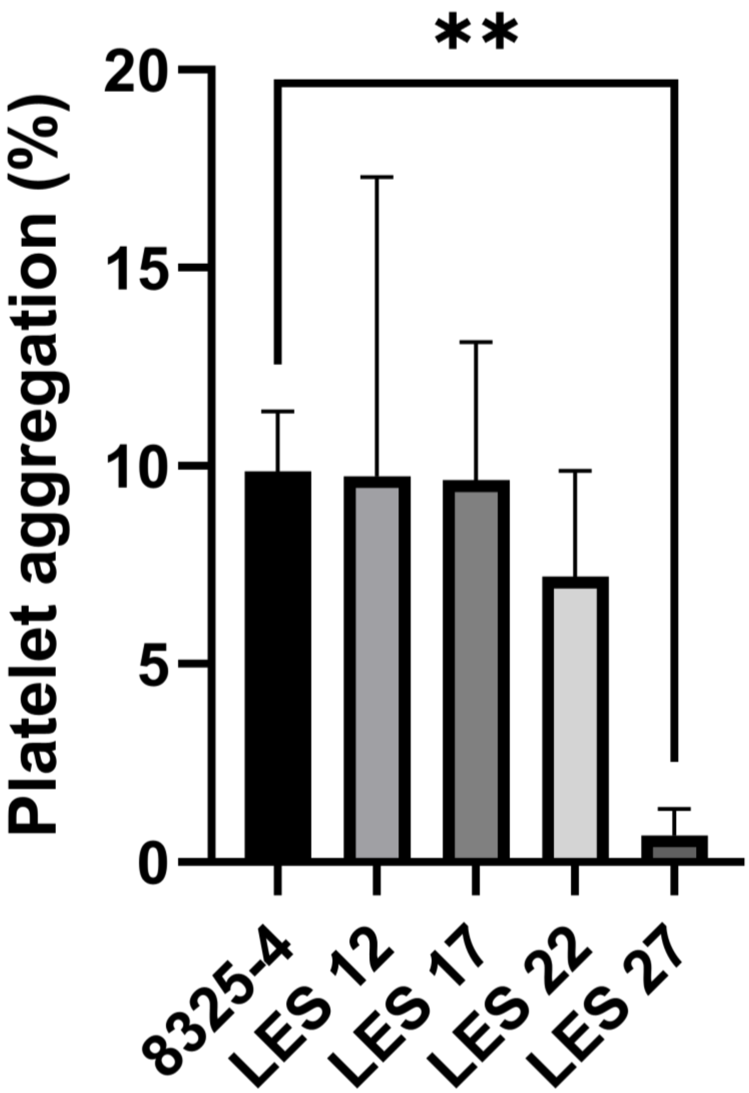

Figure S1. Aggregation of human platelets using strains of *S. aureus*. Aggregation of washed human platelets (4 x 10^8 cells/mL) stimulated with supernatant of *S. aureus* 8325-4, LES12, LES17 and LES22. Aggregation was measured as change in light transmission for 300s. Mean values $\pm$SEM. n=3, **P<0.01

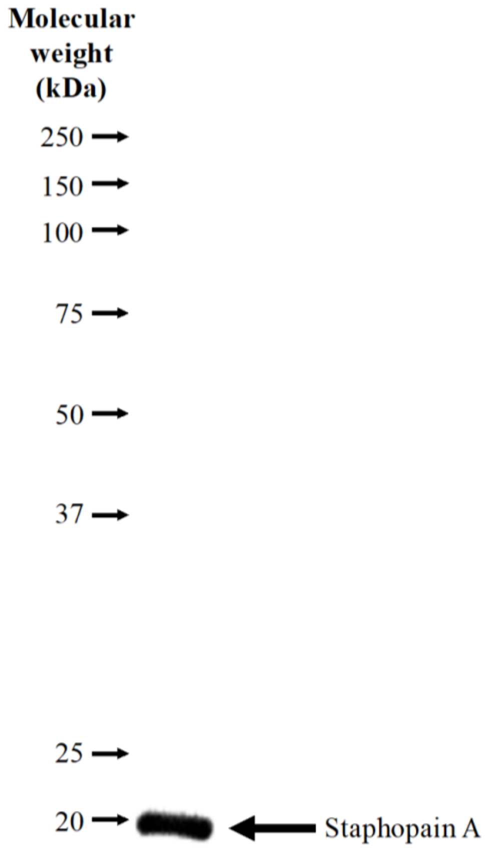

Figure S2. Purified staphopain A. Coomassie blue-stained 12% (wt/vol) SDS-PAGE gel of staphopain A purified from the supernatant of *S. aureus* DU1090.
